# Supplementary material for: ATF5 is a regulator of exercise-induced mitochondrial quality control in skeletal muscle
Source: Mol Metab. 2022 Nov 1;66:101623. doi: 10.1016/j.molmet.2022.101623 (PMC9661517; doi:10.1016/j.molmet.2022.101623)
Supplement: Multimedia component 1 [file mmc1.docx]

**Supplemental**

**Table S1.**

| Gene | Forward Primer | Reverse Primer |
| --- | --- | --- |
| ***WT (geno)*** | 5'-GGC TGG CTG GTC ACT TGT C-3' | 5'-GTC CCT GAG GAC TGT GCT TTA TC-3' |
| ***ATF5 mut (geno)*** | 5'-GCA GCC TCT GTT CCA CAT ACA CTT CA-3' | 5'-CAG AGT GGC TTC CTG CTT TAT-3' |
| ***PGC-1⍺*** | 5'-TTC CAC CAA GAG CAA GTA T-3' | 5'-CGC TGT CCC ATG AGG TAT T-3' |
| ***COX-IV*** | 5'-CTC CAA CGA ATG GAA GAC AG-3' | 5'-TGA CAA CCT TCT TAG GGA AC-3' |
| ***ATF5*** | 5'-TGG AGC GGG AGA TCC AGT A-3' | 5'-GAC GCT GGA GAC AGA CGT ACA-3' |
| ***ATF4*** | 5'-GCC GGT TTA AGT TGT GTG CT-3' | 5'-CTG GAT TCG AGG AAT GTG CT-3' |
| ***CHOP*** | 5'-CAC CAC ACC TGA AAG CAG AA-3' | 5'-AGG TGA AAG GCA GGG ACT CA-3' |
| ***HSP60*** | 5'-CTG GGT GCA AGA GCC ATA TA-3' | 5'-GAA AGG CTG CTT CTG AAC TCT-3' |
| ***mtHSP70*** | 5'-TGG CTA TTA CTG CGG GTT CT-3' | 5'-CAT CTG CTC CAC CTC CTC T-3' |
| ***LONP1*** | 5'-CGA CTT GCA CAG CCC TAT GT-3' | 5'-CGA ATG TTC CCG TAT GGT AGA T-3' |
| ***ClpP*** | 5'-CACACCAAGCAGAGCCTACA-3' | 5'-CCCAGCAGAGGAAGTTTCAG-3' |
| ***GAPDH*** | 5'-AAC ACT GAG CAT CTC CCT CA-3' | 5'-GTG GGT GCA GCG AAC TTT AT-3' |
| ***β-2Microglobulin*** | 5'-GGT CTT TCT GGT GCT TGT CT-3' | 5'-TAT GTT CGG CTT CCC ATT CT-3' |

List of primer oligonucleotide sequences used in PCR (genotyping) and real-time qPCR for *Mus Musculus.*

**Table S2.**

| **Antibody** | **Manufacturer** | **Catalogue #** |
| --- | --- | --- |
| **⍺-tubulin** | Calbiochem (Millipore) | CP06-100µg |
| **H2B** | Cell Signaling | 2934S |
| **GAPDH** | Abcam | ab8245 |
| **Aciculin** | Made in-house | N/A |
| **PFK-1** | Santa Cruz | sc-166722 |
| **LDHA** | Cell Signaling | 2012S |
| **CPT1b** | Proteintech | 22170-1-AP |
| **ATG7** | Sigma | A2856 |
| **Beclin-1** | Cell Signaling | 3738S |
| **LC3** | Cell Signaling | 4108S |
| **Parkin** | Cell Signaling | 4211S |
| **LAMP1** | Abcam | ab24170 |
| **V-ATPase** | Santa Cruz | sc-55544 |
| **NQO1** | Abcam | ab34173 |
| **HO-1** | Abcam | ab13248 |
| **GPx1** | Abcam | ab22604 |
| **MnSOD** | Upstate Cell Signaling Solutions | 06-984 |
| **Cytochrome c** | BD Biosciences | 556433 |
| **p-SAPK/JNK (T183)** | Cell Signaling | 4668S |
| **t-SAPK/JNK** | Cell Signaling | 9252T |
| **p-eIF2⍺ (S51)** | Invitrogen | 44728G |
| **t-eIF2⍺** | Cell Signaling | 9722S |
| **ATF5** | Abcam | ab184923 |
| **ATF4/CREB2** | Santa Cruz Biotechnology | sc-390063 |
| **CHOP** | Cell Signaling | 2895S |
| **VDAC1/Porin** | Abcam | ab14734 |
| **PGC-1⍺** | EMD Millipore | AB3242 |
| **LONP1** | Cell Signaling | 28020S |
| **HSP60** | Enzo Life Sciences | ADI-SPA-806-D |
| **mtHSP70** | Enzo Life Sciences | ADI-SPS-825-F |
| **Cpn10** | Enzo Life Sciences | ADI-SPA-110-D |
| **Anti-Mouse HRP-linked 2°** | Cell Signaling | 7076S |
| **Anti-Rabbit HRP-linked 2°** | Cell Signaling | 7074S |

List of antibodies used in Western Blotting.

**Fig. S1**

**Figure S1. Tissue weights of WT and ATF5 KO mice.** Various tissues, including gastrocnemius (Gastroc), soleus, heart and epididymal (Epi) fat were collected from WT and ATF5 KO mice at four months of age. Tissues were subsequently weighed and normalized for individual body weights, expressed in mg/g. Gastroc, soleus, heart (n=16-17); Epi fat, males only (n=8-9). *P<0.05, unpaired t-test.

**Fig. S2**

**
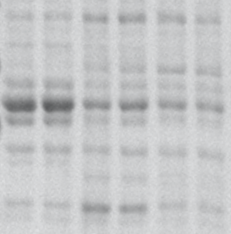
**

Gastroc

Soleus

Heart

37

ATF5

Ponceau

75

WT


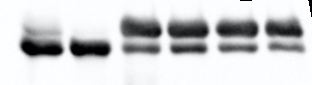


25

**B**

**A**

**Figure S2. ATF5 protein content in striated muscles of WT mice. A)** Western blot of ATF5 in whole muscle lysates extracted from gastrocnemius (Gastroc), soleus, and heart tissue with the corresponding Ponceau stain. **B)** Quantification of ATF5 protein expression corrected for Ponceau (n=4). The region between 75 and 25 kDa in the Ponceau stain was used to correct for minor variations in loading across tissues. A.U., arbitrary units. *P<0.05, One-way ANOVA, post-hoc analysis.

**Figure S3.**

PGC-1$\alpha$ siRNA

100

NTC

SCR

WM


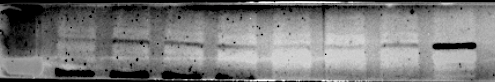

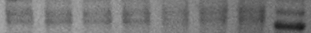


Myotubes

Ponceau

PGC-1$\alpha$

**Figure S3. Verification of the PGC-1**$\boldsymbol{\alpha}$ **band in C2C12 myotubes transfected with a PGC-1**$\boldsymbol{\alpha}$ **siRNA.** Western blot of PGC-1$\alpha$ in SCR or PGC-1$\alpha$ siRNA-transfected myotubes with the corresponding Ponceau stain. Three replicates from SCR and siRNA conditions are shown, with a non-transfected control (NTC) cell extract and a whole muscle (WM) tissue lysate run on the same gel.
